# Clinical, Bacteriological, and Genetic Characterization of Bone and Joint Infections Involving Linezolid-Resistant Staphylococcus epidermidis: a Retrospective Multicenter Study in French Reference Centers

**DOI:** 10.1128/spectrum.04190-22

**Published:** 2023-05-03

**Authors:** François Coustillères, Victor Renault, Stéphane Corvec, Céline Dupieux, Patricia Martins Simões, Marie Frédérique Lartigue, Chloé Plouzeau-Jayle, Didier Tande, Claudie Lamoureux, Carole Lemarié, Rachel Chenouard, Frédéric Laurent, Adrien Lemaignen, Pascale Bémer, the CRIOGO (Centre de Référence des Infections Ostéo-articulaires du Grand Ouest) Study Team

**Affiliations:** a Service des Maladies Infectieuses, Centre Hospitalier Régional Universitaire, Centre Régional de Référence pour la prise en charge des IOA complexes (CRIOGO), Tours, France; b Service de Bactériologie et des Contrôles microbiologiques, Hôtel-Dieu, Centre Hospitalier Universitaire, Centre Régional de Référence pour la prise en charge des IOA complexes (CRIOGO), Nantes, France; c Hospices Civils de Lyon, Institut des Agents Infectieux, Service de Bactériologie, Centre National de Référence des Staphylocoques, Centre Régional de Référence pour la prise en charge des IOA complexes (CRIOAC Lyon), France; d Service de Bactériologie et d’Hygiène, Centre Hospitalier Régional Universitaire, Centre Régional de Référence pour la prise en charge des IOA complexes (CRIOGO), Tours, France; e Service de Bactériologie et d’Hygiène, Centre Hospitalier Universitaire, Centre Régional de Référence pour la prise en charge des IOA complexes (CRIOGO), Poitiers, France; f Service de Bactériologie et d’Hygiène, Centre Hospitalier Universitaire, Centre Régional de Référence pour la prise en charge des IOA complexes (CRIOGO), Brest, France; g Service de Bactériologie et d’Hygiène, Centre Hospitalier Universitaire, Centre Régional de Référence pour la prise en charge des IOA complexes (CRIOGO), Angers, France; Centre Hospitalier Universitaire d’Angers: Pierre Abgueguen; Centre Hospitalier Universitaire d’Angers: Pierre Abgueguen; Centre Hospitalier Universitaire d’Angers: Pierre Abgueguen; Centre Hospitalier Universitaire d’Angers: Pierre Abgueguen; Centre Hospitalier Universitaire de Brest; Centre Hospitalier Universitaire de Brest; Centre Hospitalier Universitaire de Brest; Centre Hospitalier Universitaire de Brest; Centre Hospitalier Universitaire de Brest; Centre Hospitalier Universitaire de Brest; Centre Hospitalier Universitaire de Nantes; Centre Hospitalier Universitaire de Nantes; Centre Hospitalier Universitaire de Nantes; Centre Hospitalier Universitaire de Nantes; Centre Hospitalier Universitaire de Nantes; Centre Hospitalier Universitaire de Nantes; Centre Hospitalier Universitaire de Nantes; Centre Hospitalier Universitaire de Nantes; Centre Hospitalier Universitaire de Nantes; Centre Hospitalier Universitaire de Nantes; Centre Hospitalier Universitaire de Nantes; Centre Hospitalier Universitaire de Poitiers; Centre Hospitalier Universitaire de Poitiers; Centre Hospitalier Universitaire de Poitiers; Centre Hospitalier Universitaire de Poitiers; Centre Hospitalier Universitaire de Poitiers; Centre Hospitalier Universitaire de Rennes; Centre Hospitalier Universitaire de Rennes; Centre Hospitalier Universitaire de Rennes; Centre Hospitalier Universitaire de Rennes; Centre Hospitalier Universitaire de Rennes; Centre Hospitalier Régional Universitaire de Tours; Centre Hospitalier Régional Universitaire de Tours; Centre Hospitalier Régional Universitaire de Tours; Centre Hospitalier Régional Universitaire de Tours; Centre Hospitalier Régional Universitaire de Tours; Centre Hospitalier Régional Universitaire de Tours; Centre Hospitalier Régional Universitaire de Tours; Taichung Veterans General Hospital

**Keywords:** linezolid-resistant multidrug-resistant *Staphylococcus epidermidis*, bone and joint infections, linezolid exposure, sequence type ST2, multiresistance, resistance to linezolide, *Staphylococcus epidermidis*

## Abstract

The choice of the best probabilistic postoperative antibiotics in bone and joint infections (BJIs) is still challenging. Since the implementation of protocolized postoperative linezolid in six French referral centers, linezolid-resistant multidrug-resistant Staphylococcus epidermidis (LR-MDRSE) strains were isolated in patients with BJI. We aimed here to describe clinical, microbiological, and molecular patterns associated with these strains. All patients with at least one intraoperative specimen positive for LR-MDRSE between 2015 and 2020 were included in this retrospective multicenter study. Clinical presentation, management, and outcome were described. LR-MDRSE strains were investigated by MIC determination for linezolid and other anti-MRSA antibiotics, characterization of genetic determinants of resistance, and phylogenetic analysis. Forty-six patients (colonization *n* = 10, infection *n* = 36) were included in five centers, 45 had prior exposure to linezolid, 33 had foreign devices. Clinical success was achieved for 26/36 patients. Incidence of LR-MDRSE increased over the study period. One hundred percent of the strains were resistant to oxazolidinones, gentamicin, clindamycin, ofloxacin, rifampicin, ceftaroline, and ceftobiprole, and susceptible to cyclins, daptomycin, and dalbavancin. Susceptibility to delafloxacin was bimodal. Molecular analysis was performed for 44 strains, and the main mutation conferring linezolid resistance was the 23S rRNA G2576T mutation. All strains belonged to the sequence type ST2 or its clonal complex, and phylogenetic analysis showed emergence of five populations corresponding geographically to the centers. We showed the emergence of new clonal populations of highly linezolid-resistant S. epidermidis in BJIs. Identifying patients at risk for LR-MDRSE acquisition and proposing alternatives to systematic postoperative linezolid use are essential.

**IMPORTANCE** The manuscript describes the emergence of clonal linezolid-resistant strains of Staphylococcus epidermidis (LR-MDRSE) isolated from patients presenting with bone and joint infections. Incidence of LR-MDRSE increased over the study period. All strains were highly resistant to oxazolidinones, gentamicin, clindamycin, ofloxacin, rifampicin, ceftaroline, and ceftobiprole, but were susceptible to cyclins, daptomycin, and dalbavancin. Susceptibility to delafloxacin was bimodal. The main mutation conferring linezolid resistance was the 23S rRNA G2576T mutation. All strains belonged to the sequence type ST2 or its clonal complex, and phylogenetic analysis showed emergence of five populations corresponding geographically to the centers. LR-MDRSE bone and joint infections seem to be accompanied by an overall poor prognosis related to comorbidities and therapeutic issues. Identifying patients at risk for LR-MDRSE acquisition and proposing alternatives to systematic postoperative linezolid use become essential, with a preference for parenteral drugs such as lipopeptids or lipoglycopeptids.

## INTRODUCTION

The medical-surgical management of bone and joint infections (BJIs) is often complicated by the presence of devices, comorbidities, or allergies in patients and the presence of polymicrobial infections or antibiotic-resistant bacteria. To address this problem, the French Ministry of Health has created 30 reference centers throughout the country since 2008, called Centers de Référence pour les Infections Ostéo-Articulaires complexes (CRIOAc). These centers provide multidisciplinary expertise in the management of complex BJIs, including weekly meetings held in each reference center. Clinical data from CRIOAc were reported for the first time in 2021 (1). Among the infectious episodes analyzed from 2014 to 2019, 61% (17,328/28,365) were defined as complex, occurring in patients with more than two comorbidities for one-third of them. Resistant bacteria were isolated in 20.3% (3977/19,574) of documented infections and were significantly associated with a foreign device (22.1% *versus* 17.8%, *P* < 0.001) and multiple infections multiple mechanisms and/or multiple sites (37.2% *versus* 19.9%, *P* < 0.001).

One of the challenges for the management of these complex infections is the choice of early probabilistic broad-spectrum antibiotic therapy in the immediate postoperative period targeting methicillin-resistant staphylococci and group III Enterobacterales. Although not positioned as a first-line treatment in French recommendations (2), the six centers of Angers, Brest, Nantes, Poitiers, Rennes, and Tours forming the Centre de Référence des Infections Ostéoarticulaires du Grand Ouest (CRIOGO) have chosen linezolid along with piperacillin-tazobactam for this indication in 2015 for the first center, 2017 for three centers, and 2019 for the last two.

The oxazolidinone class presents several properties justifying its prescription: broad spectrum on the Gram-positive cocci involved (3), good bone diffusion (4), inhibitory property on biofilm formation (5, 6), as well as a number of advantages compared to its usual alternative, vancomycin: good immediate tolerance with less nephrotoxicity, and ease of use, with excellent oral bioavailability (100%), not requiring a central line nor adaptation of the dosage to weight, renal, and hepatocellular functions (7, 8).

Since its marketing in 2002, the gradual increase in hospital prescription of linezolid has led to the emergence of resistance, mainly in Staphylococcus epidermidis species (9, 10). In staphylococci, this acquisition involves various mechanisms regularly coexpressed (11, 12): mutation(s) in the 23S ribosomal target or in the associated L3 -L4 -L22 proteins, and acquisition of plasmids carrying the *cfr* gene, which is responsible for a methylation of the target. Linezolid-resistant phenotype is most often associated with multidrug resistance, directly related to the mechanism involved, or to the combination with other resistance mechanisms (13). It may be subject to clonal epidemic dissemination as already described in several hospital departments, including French ones (14–19).

Comparative and functional genomics of S. epidermidis characterized three multidrug-resistant S. epidermidis (MDRSE) lineages emerging in the 1980s and spreading in 24 countries around the world, including France (20). These strains are resistant to rifampicin by acquisition of specific *rpoB* mutations (20). This study described a low percentage (<10%) of linezolid-resistant strains (LR-MDRSE), which were limited to Europe (Germany and France); this resistance was mainly mediated by ribosomal mutations and more rarely by the presence of *cfr*, only identified in German isolates (20).

Among the six CRIOGO's centers, the first case of acquisition of a LR-MDRSE strain was reported by the center of Nantes that introduced linezolid in 2015, in a patient managed for BJI, multioperated and preexposed to linezolid (21). Since then, a recent study showed the persistence and spread of a *cfr*-carrying plasmid in the same center from 2015 to 2018, related to the recently reported LR-MDRSE ST2 lineage (22).

The objective of the present retrospective multicenter study was therefore to analyze the LR-MDRSE isolated from osteoarticular samples, whether these strains were colonizing or infecting. The clinical characteristics of patients, their previous exposure to linezolid, and their medical-surgical management were analyzed. The genetic resistance determinants were characterized, and a genomic comparative analysis of those isolates was performed. Finally, therapeutic alternatives were proposed by testing the susceptibility of LR-MDRSE strains to the most recent antibiotic molecules.

## RESULTS

### Clinical results.

In this study, 46 patients with at least one LR-MDRSE isolated strain were retrospectively included, and their characteristics are presented in Table 1 (median age: 71 years; sex ratio F/M: 16/30). The majority had at least one comorbidity, including cancer and obesity. Thirty-three (71.7%) patients underwent orthopedic surgery on foreign device, 15 (32.6%) on joint prosthesis, and 7 (15%) on spacer. Surgical management of the 46 LR-MDRSE infected or colonized patients was detailed in Table S1.

**TABLE 1:** Clinical and microbiological characteristics of patients infected or colonized by LR-MDRSE^*a*^

	Total	Infection	Colonization
Characteristics	*N* (%)	*N* (%)	*N* (%)
Age (yrs old)	70.5	71.6	66.6
Gender (F/M)	16/30	12/24	4/6
wt (kg)	78	-	-
Center			
1	3 (6.5)	3 (8.3)	0 (0)
2	5 (10.9)	5 (13.9)	0 (0)
3	17 (37.0)	13 (36.1)	4 (40.0)
4	10 (21.7)	8 (22.2)	2 (20.0)
5	11 (23.9)	7 (19.4)	4 (40.0)
Illness	37 (80.4)	-	-
Active cancer or remission for less than 5 yrs	11 (23.9)	-	-
Immunocompromised	6 (13.0)	-	-
Diabetes	14 (30.4)	-	-
Obesity (BMI > 30kg/m² body surface)	21 (45.7)	-	-
Foreign device	33 (71.7)	29 (80.6)	4 (40.0)
Joint prosthesis	15 (32.6)	14 (38.9)	1 (10.0)
Spacer	7 (15.2)	7 (19.4)	0 (0)
Osteosynthesis	5 (10.9)	3 (8.3)	2 (20.0)
External fixation device	3 (6.5)	2 (5.6)	1 (10.0)
Other arthrodesis device	2 (4.3)	2 (5.6)	0 (0)
Masquelet	1 (2.2)	1 (2.8)	0 (0)
No device	13 (28.2)	7 (19.4)	6 (60.0)
Past removed device on the same anatomical site	6 (13.0)	3 (8.3)	3 (30.0)
Osteitis – no past or present device	7 (15.2)	4 (11.1)	3 (30.0)
Pelvis/Spine bones	3 (6.5)	1 (2.8)	2 (20.0)
Tibia/Femur	2 (4.3)	2 (5.5)	0 (0)
Hand	1 (2.2)	0 (0)	1 (10.0)
Diabetic foot	1 (2.2)	1 (2.8)	0 (0)
Past bone and joint infections	44 (95.7)	35 (97.2)	9 (90.0)
1	25 (54.3)	21 (58.3)	4 (40.0)
≥2	19 (41.3)	14 (38.9)	5 (50.0)
Including linezolid susceptible S. epidermidis	7 (15.2)	7 (19.4)	0 (0)
Prior hospital antibiotics (excluding prophylaxis)	45 (97.8)	-	-
Oxazolidinone	45 (97.8)	-	-
Beta-lactam	45 (97.8)	-	-
Fluoroquinolone	26 (56.5)	-	-
Rifampicin	18 (39.1)	-	-
Microbiological findings			
no. of positive samples ≥2	34 (73.9)	32 (88.9)	2 (20.0)
Other associated pathogen(s)	27 (58.7)	21 (58.3)	6 (60.0)
Total	46 (100)	36 (100)	10 (100)

a Age and weight: median values. BMI: body mass index.

**FIG 1:**
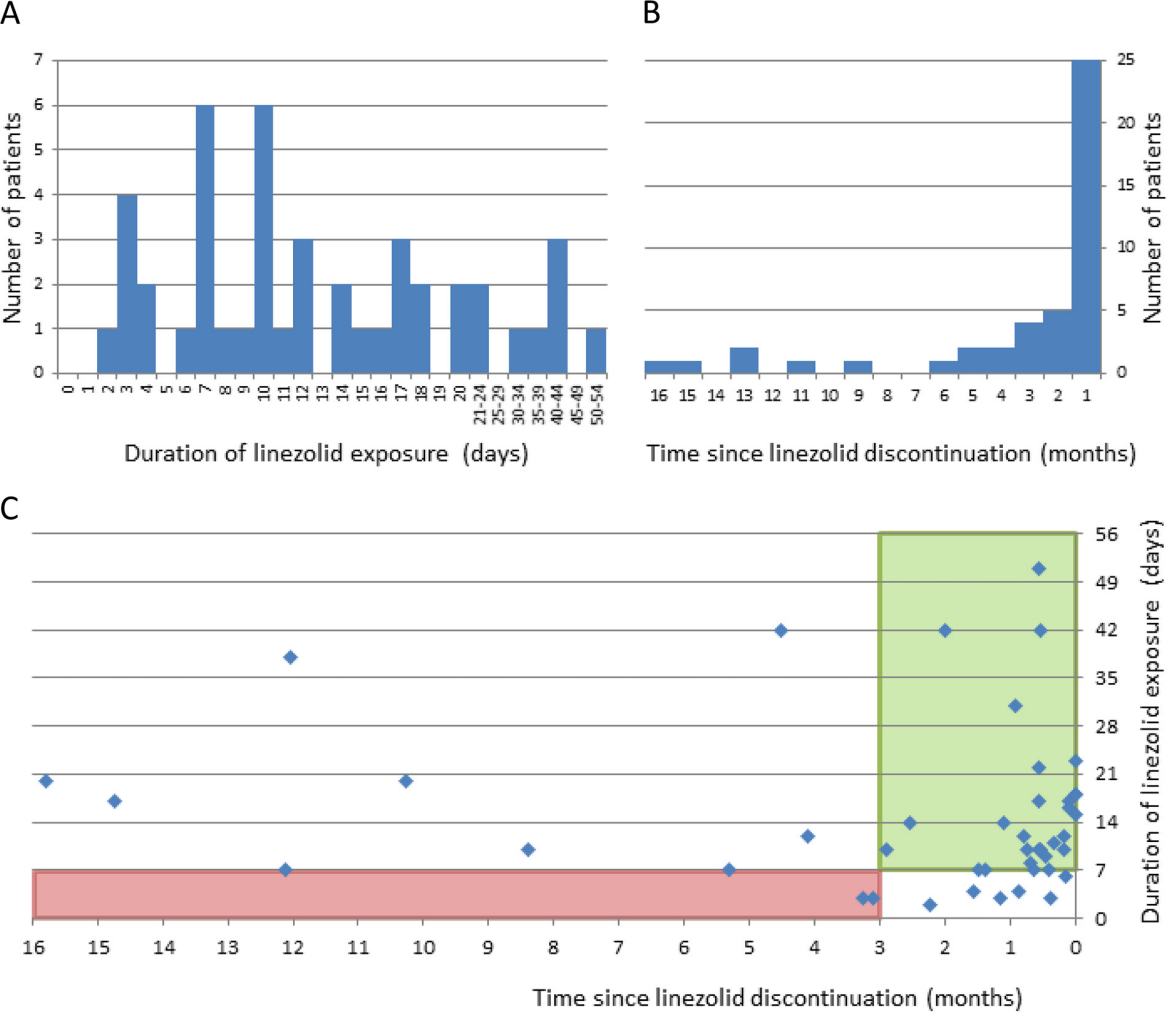
Linezolid exposure in patients infected or colonized with LR-MDRSE (*n* = 45). (A) Duration of linezolid exposure prior to LR-MDRSE isolation (median: 11.0 days). (B) Time from end of linezolid use to identification of a LR-MDRSE isolate (median: 0.8 months). (C) Duration/time of exposure diagram for each infected or colonized patient. The green rectangle corresponds to an exposure of more than 1 week within 3 months before LR-MDRSE isolation, the red rectangle to an exposure of less than 1 week and a delay of more than 3 months since discontinuation.

Prior linezolid exposure was found in all patients except one. This 62-year-old patient presented with acute sepsis 6 days after surgery for chondrosarcoma. All five lavage samples were positive in culture for LR-MDRSE and E. cloacae strains. The patient did not receive linezolid antibiotic prophylaxis. For the remaining patients, linezolid had been prescribed postoperatively as a probabilistic treatment in 78% of cases and continued after species identification in 17%. Linezolid was also prescribed during a nonorthopedic infectious episode in three patients (7%) admitted to the ICU. The duration and timing of linezolid exposure was highly variable (Fig. 1). For 14 (30%) patients, resistance was identified after a course ≤7 days consistent with a linezolid probabilistic use (Fig. 1A) and for 11 (24%) of them, LR-MDRSE was identified at a distance from discontinuation >3 months (Fig. 1B). In four (9%) patients, LR-MDRSE was identified after a short duration of exposure and more than 3 months since discontinuation (Fig. 1C). A total of 18 patients had also prior exposition to rifampicin (median time from end of rifampicin use to LR-MDRSE identification: 2.2 months), all of them for an extended duration as a previous antibiotherapy for past bone and joint infection.

Thirty-six (78%) of the patients were found to have a LR-MDRSE infection, and 10 (22%) were found to be colonized (Table 1). Nearly 60% of the infected patients had one previous BJI episode and nearly 40% at least two septic episodes, seven (15.2%) of which were due to linezolid-susceptible S. epidermidis strains (Table 1). None of the patients found to be colonized had subsequent LR-MDRSE culture-positive samples. The majority (28; 80%) of LR-MDRSE infections occurred on devices, which were considered acute for 12 of them (43%), and chronic for 16 (57%, median time to management: 133 days [29:1,110]).

Surgical management of infected patients consisted of debridement, antibiotics, and implant retention (DAIR) in nine cases (25%), and complete replacement/ablation of material in 18 cases (50%), (Table 2). In 3 patients, LR-MDRSE was documented and treated during the second stage of a device removal/replacement. A total of 35 patients (97%) received LR-MDRSE targeted antibiotherapy for a median of 84 days (9; 103), of which 20 (56%) were treated with dual therapy at least in part. Most patients (23; 64%) received parenteral therapy for the whole duration of treatment. Preferred prescribing regimens were: co-trimoxazole-fusidic acid combination, ceftaroline-daptomycin, and doxycycline in mono- or dual therapy (Table 2).

**TABLE 2:** Management details for the 36 patients presenting LR-MDRSE bone and joint infection (BJI)

	Monotherapy	Dual therapy^*a*^	Total	Length (days)	
Characteristics	*N* (%)	*N* (%)	*N* (%)	Median	[min–max]
Surgical management					
Complete implant exchange or ablation			18 (50.0)		
Chronic infection			12 (33.3)		
Debridement, Antibiotics and Implant Retention (DAIR)			9 (25.0)		
Acute infection			6 (16.7)		
Debridement without device			5 (13.9)		
Surgically maintained fistula			1 (2.8)		
Amputation			2 (5.6)		
Medical management only			1 (2.8)		
Appropriate empirical antibiotics			6 (16.7)		
Antibiotics after documentation^*b*^					
Vancomycin/teicoplanin	3 (8.3)	3 (8.3)	6 (14.7)	26.5	[15–90]
Daptomycin	6 (16.7)	10 (27.7)	16 (44.4) (41.7)(41.7)	65	[3–90]
Ceftarolin	0 (0)	5 (13.9)	5 (13.9)	42	[13–105]
Doxycyclin	6 (16.7)	4 (11.1)	10 (27.8)	62	[9–90]
Co-trimoxazole	1 (2.8)	9 (25.0)	10 (27.8)	66.5	[6–84]
Fusidic acid	0 (0)	6 (16.7)	6 (16.7)	84	[9–84]
Dalbavancin	3 (8.3)	2 (5.6)	5 (13.9)	2 doses	[1–6]
Total antibiotics	15 (41.7)	20 (55.6)	35 (97.2)	84	[9-103]
Success			26 (72.2)		
Total			36 (100.0)		

a Patients who were treated at least partially with dual therapy with one of the molecules were considered in the dual therapy group for that molecule and for total antibiotic therapy. Data on effective antibiotic therapy available for 35 patients; 1 patient did not receive postoperative antibiotic therapy following a transtibial amputation but was considered infected on the basis of microbiological data (4/5 samples positive for LR-MDRSE). Duration of curative antibiotic therapy arbitrarily set at 90 days for patients who received suspensive treatment with doxycycline.

b All bioavailable antimicrobial agents were used orally as often as possible, other drugs were dispensed parenterally.

After a median follow-up of 11 months (0; 51), 26 (72%) patients were considered in remission, and 10 (28%) in failure of management (microbiological failure *n* = 4 (11%), amputation, or unplanned arthrodesis for uncontrolled sepsis *n* = 1 (3%), death within 12 months after pathogen isolation *n* = 5 (14%)) (Table 2). The death of two patients was *a priori* not directly linked to the current infection (pulmonary embolism and pericarditis for one, pulmonary embolism for the other), but a lack of infection control might have interfered as a contributing factor. One patient died of septic shock without microbial identification, probably not solely due to LR-MDRSE. For the two others, death was more clearly directly linked to the LR-MDRSE infection. The high death rate among patients infected with LR-MDRSE was correlated with their heavy comorbidities and difficult to treat infections. Long-term suppressive antibiotic therapy with doxycycline was prescribed in four patients (11%), with a favorable functional outcome after a median follow-up of 8 months.

### Microbiological results.

Among the 32 infected patients, 3 patients had two, 9 patients had three, 7 patients had four and 13 patients had five LR-MDRSE positive samples. Samples were polymicrobial in 58.3% of infections. LR-MDRSE isolates were associated with Gram-negative bacilli, staphylococci, and Cutibacterium acnes in 43%, 19%, and 14.3% of infections, respectively (for details, see Table S2).

**FIG 2:**
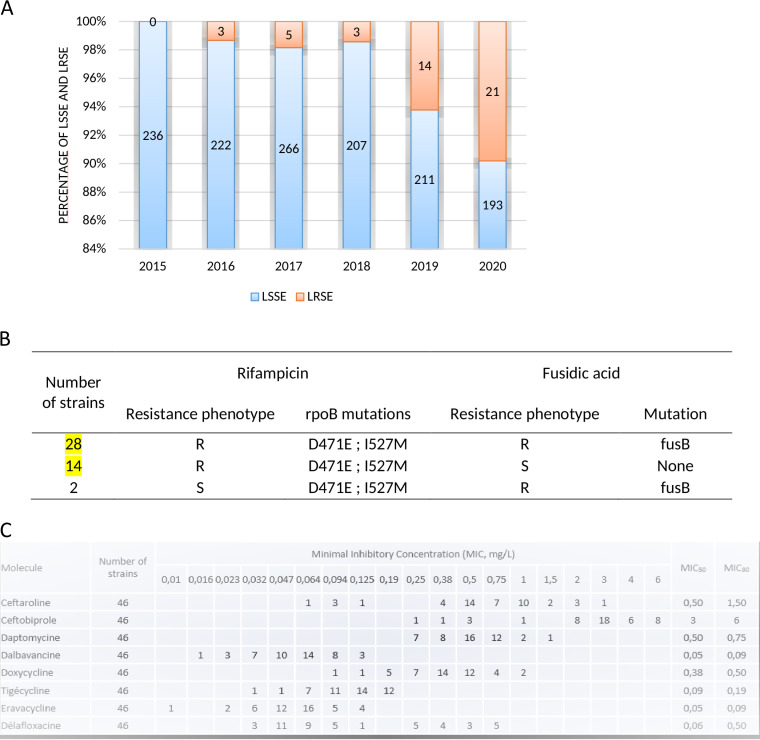
Phenotypic characterization of S. epidermidis isolates at CRIOGO’s centers over the study period. (A) Distribution of linezolid-susceptible (LSSE) and linezolid–resistant (LRSE) S. epidermidis isolates. (B) Antibiotic resistance patterns of LRSE isolates. (C) MICs distribution and MIC50/90 of ceftaroline, ceftobiprole, daptomycin, dalbavancin, tigecycline, eravacycline, and delafloxacin for LRSE isolates.

The incidence of LR-MDRSE in osteoarticular samples increased over the study period, accounting for 6% and 10% of all S. epidermidis isolates in 2019 and 2020, respectively, *versus* less than 2% from 2015 to 2018 (Fig. 2A). All LR-MDRSE strains but three were resistant to methicillin. All LR-MDRSE isolates showed a high level of resistance to linezolid and tedizolid (MICs >256 and >32 mg/L respectively), and were resistant to gentamicin, clindamycin, and ofloxacin, but susceptible to cyclins. The strains in our study appeared to be susceptible to vancomycin (MIC90 ≤1 mg/L), probably corresponding to strains with decreased susceptibility to glycopeptides, which are difficult to detect by routine laboratory methods. Isolates harbored two main resistance patterns to other antibiotics, one resistant to rifampicin and fusidic acid (61%), and the second resistant to rifampicin and susceptible to fusidic acid (33%) (Fig. 2B).

The MIC90s of ceftaroline and even more of ceftobiprole, were above the resistance threshold according to CASFM/EUCAST 2021 (23). The MIC90s of daptomycin, dalbavancin, tigecycline, and eravacycline were still below the resistance breakpoints according to CASFM/EUCAST. For delafloxacin, the MICs revealed a bi-modal distribution, with 63% of susceptible isolates, and 37% resistant (Fig. 2C).

### Molecular results for the 44 MRSE-LR strains.

The G2576T mutation in 23S rRNA was identified in 42/44 strains, and the T2504A in two strains. A total of 27/44 strains harbored also the three mutations L101V-Q136L-M156T in the *rplC* gene coding for the L3 ribosomal protein. L4 ribosomal protein was mutated in G69R for 21/44 (47.7%) strains. No mutation was detected in the L22 ribosomal. Six (6/44, 13.6%) strains from Nantes harbored the *cfr* gene.

All 44 strains harbored the two *rpoB* mutations (D471M and I527M) conferring a cross-resistance to rifampicin and vancomycin. The *fusB* gene was identified in all the 30 strains resistant to fusidic acid.

Concerning the resistance to fluoroquinolones, all except one strain had the double mutation S80F-D84Y in the *parC* gene. All except one strain with a delafloxacin MIC below 0.125 mg/L harbored the single mutation S84Y in *gyrA*, while all the strains with a MIC >0.125 had the double mutation S84F-E88K in *gyrA*. No mutation was observed in *parE*.

All the strains except two belonged to the sequence type 2 (ST2 *n* = 42, ST2-like *n* = 1, ST87 *n* = 1).

**FIG 3:**
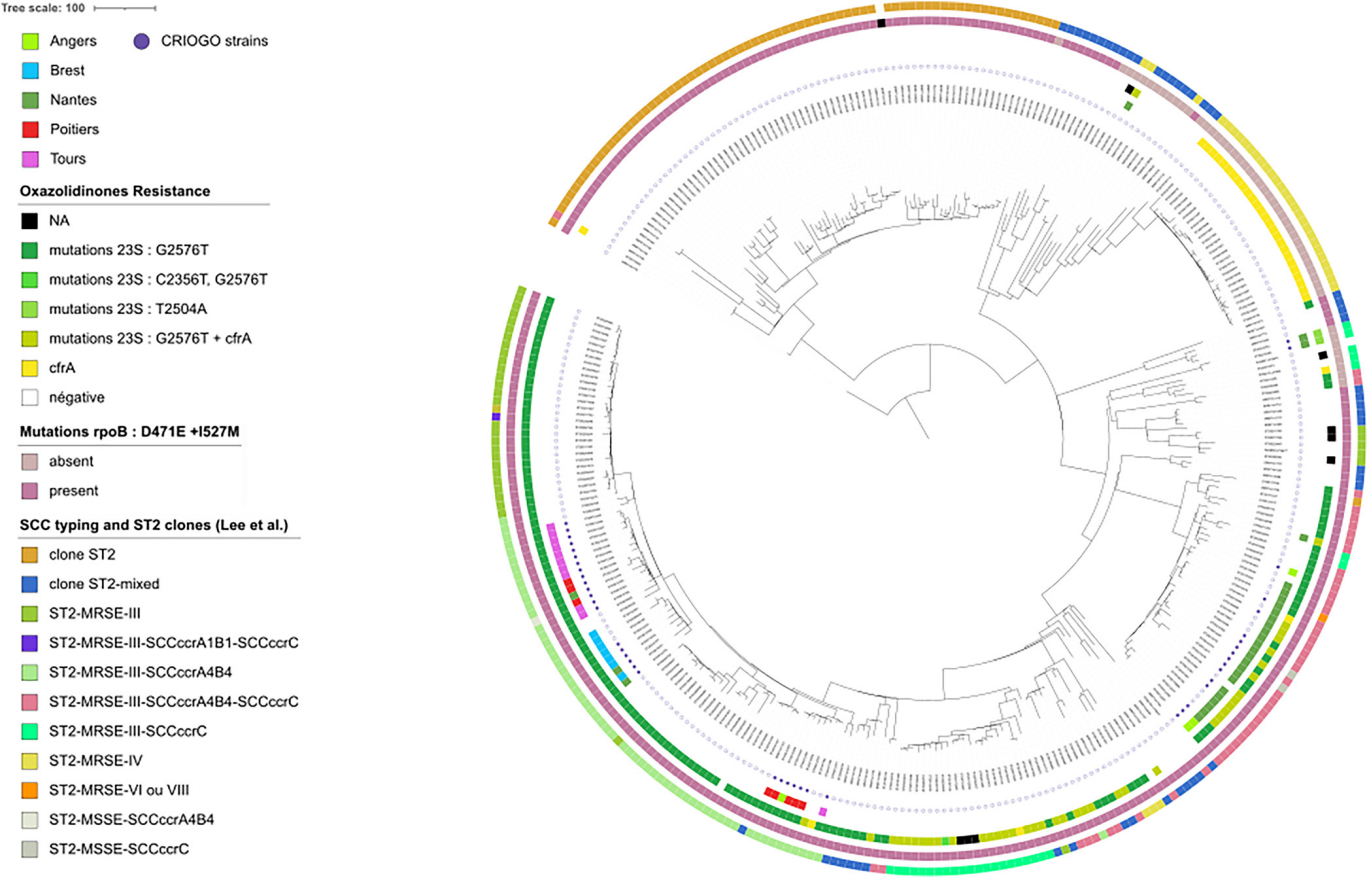
Maximum likelihood phylogenetic tree of 348 ST2 S. epidermidis isolates based on 7271 core genome SNPs. The 44 S. epidermidis form the CRIOGO study are indicated by purple circles in the first metadata inner circle. The second metadata inner circle indicates samples from the 5 cities participating in the CRIOGO study. The third metadata inner circle indicates the linezolid associated resistance genotype. The fourth metadata inner circle indicates the presence or absence of the 2 *rpoB* point mutations previously described by Lee et al. (20), to be associated with cross-resistance to vancomycin. The fifth metadata inner circle indicates the SCC typing, ST2, and ST2-mixed clones.

Phylogenetic analysis showed the emergence of five populations form a recent common ancestor (Fig. 3): the “Tours” population (group with a predominance of strains from Tours), the “Brest” population (predominance of strains from Brest), the “Poitiers” population (predominance of strains from Poitiers), the “Nantes 1” and “Nantes 2” populations (predominance of strains from Nantes). Seven strains belong to a group different from their city of origin, suggesting patient movement in these regions (Fig. 3).

## DISCUSSION

This study characterized the emergence of LR-MDRSE when using probabilistic treatment with linezolid for BJIs within five CRIOGO hospitals. The majority of BJIs occurred on devices, in patients exposed to linezolid, with at least one comorbidity and a history of osteoarticular sepsis. These strains showed multidrug resistance to oral antibiotics used to treat BJIs. Resistance to oxazolidinones was mainly mediated by target mutations. Comparative genomic analysis suggested the emergence of new clonal lineages/populations in each different hospital. This suggests a scenario of patient’s cross contamination within each hospital and few exchanges between hospitals.

In the early 2000s, isolation of LR-MDRSE strains was rare in the antibiotic resistance surveillance programs (24, 25). Subsequently, these strains emerged in several European hospitals (14–19). The ST2 clone was predominant and could become endemic in the long term (14–16). In France, the first linezolid-resistant S. epidermidis strains were isolated from bacteremia, catheter-related infections, deep infections, or respiratory-tract infections in immunocompromised patients (17). Most studies have shown a correlation between the emergence of these strains and the increasing use of linezolid, which resulted in substantial selection pressure in favor of LR-MDRSE strains (14, 17).

Our study is the first concerning complex BJIs on device in patients with risk factors and a history of sepsis. Almost all patients had been exposed to linezolid but preexposure to linezolid was highly variable, with the emergence of a resistant strain after a short exposure ≤7 days in 30% of cases. In our centers, many patients with recurrent or relapsed device infection have already been exposed to linezolid at the time of reoperation, in particular, because of a first episode of orthopedic infection initially treated by a probabilistic combination, including linezolid. It is therefore difficult to predict which patients are at risk and for whom the postoperative probabilistic antibiotic therapy should include an alternative antibiotic to linezolid.

The CRIOGO centers have solid experience of linezolid use in the treatment of device infections. Animal studies demonstrated its efficacy in the treatment of BJIs due to Gram-positive cocci (26, 27), and clinical data showed satisfactory efficacy in the treatment of device infections (28). The incidence of LR-MDRSE carriage appears to be directly related to linezolid consumption in health care facilities (14–19), and reducing consumption of oxazolidinones could help limit this incidence (17). Controlling the consumption of linezolid will allow for the preservation of its efficacy and its continued use, either as a probabilistic treatment or as a targeted treatment for BJIs due to Gram-positive resistant bacteria. Its exclusive prescription in hospitals makes it easier to control its use in France.

The high frequency of methicillin-resistant staphylococci as BJI pathogens (31% [3],) makes coverage of these strains necessary in probabilistic care. Other CRIOACs used alternative combinations (ceftriaxone/cefepime–vancomycin/daptomycin) for the postoperative probabilistic treatment of device infection. Ceftobiprole was chosen in this indication, with no resistance identified so far in any of the Staphylococcus species strains tested (29). However, our study showed an elevation of ceftobiprole MICs toward resistance in these LR-MDRSE strains. MICs for ceftobiprole were also high for LR-MDRSE strains isolated from bacteremia and catheter infections, prompting caution in the use of these molecules (30).

The prognosis of infected patients was burdened by severe comorbidities and therapeutic difficulties related to the LR-MDRSE strains and to the other difficult-to-treat isolated bacteria, such as Gram-negative bacilli. The majority of infected patients underwent surgery with removal or complete replacement of the infected device. Tedizolid was not an alternative, because of a high-level resistance in all strains. LR-MDRSE remained susceptible for parenteral drugs, with widespread use of dalbavancin and the combination of daptomycin and ceftaroline. The success rate (72%) was nevertheless little different from that usually encountered in the management of complex periprosthetic BJIs, but it should be emphasized that our criteria for failure were relatively narrow. Thus, relapse to another germ or orthopedic reoperation without identification of the LR-MDRSE strain was not considered a failure.

CRIOGO strains mostly (42/44, 95%) belonged to the ST2 clonal complex, and express chromosomal mutations in 23S RNA, L3, and L4 proteins conferring high cross-resistance to linezolid and tedizolid as previously described (14, 17). The *cfr* gene was found in only 13.3% (*n* = 6) of strains, exclusively in the Nantes center and to one of the two populations found in this center. According to literature data, plasmid resistance to linezolid was heterogeneous, either absent (14, 15) or present in 19% (17) to 63% (18) of strains. The global spread of three multidrug-resistant S. epidermidis clones (ST2, ST2-mixed lineage, ST23) highly resistant to rifampin has been described in 96 hospitals in 24 countries (20). Recently, the global MDR S. epidermidis ST2 lineage has been found to be common in S. epidermidis from prosthetic joint infections (31–33) and was significantly associated with strong biofilm production (34). In our study all strains contained both mutations D471E and I527M conferring high level resistance to rifampicin. Resistance to rifampicin was reported to be significantly higher in strains from patients with treatment failure, supporting the results from previous studies where inadequate rifampicin regimens led to worse outcomes (35). Very recently, the majority of the rifampicin resistant strains showed a strong biofilm phenotype, belonged mainly to the ST2 group, and were associated with treatment failure (34). The five CRIOGO populations are found within the “ST2-mixed” clade described (20), suggesting the emergence of new clonal lineages within an ancestral population. The correlation was almost perfect between clonal lineages and corresponding hospitals, suggesting the emergence of nosocomial lineages independently in each hospital. Displacement of patients from their region of origin was confirmed for at least one patient.

The main strength of our clinical study is to focus on BJIs managed in five different specialized centers, during weekly multidisciplinary meetings allowing us to evaluate patients’ follow-up and evolution.

The limitations of this study are its retrospective nature, with a certain number of missing data, the risk of classification bias inherent with this methodology, a small number of patients, and heterogeneity of the population included (infections/colonizations, presence or absence of device, a broad range of surgical methods were included). The data were collected in full by the principal investigator for all the centers, which nevertheless made it possible to homogenize the data collection method.

The main significance of our study is the emergence of new clonal populations of multidrug-resistant S. epidermidis in BJIs. Therefore, parenteral use of molecules such as daptomycin or dalbavancin is required. Concerning delafloxacin, it seems reasonable to limit its use for strains with MICs ≤0.125 mg/L.

The incidence of LR-MDRSE in BJIs is an epidemiological indicator to be monitored especially for centers using linezolid as a postoperative probabilistic treatment. LR-MDRSE BJI seem to be accompanied by an overall poor prognosis. Identifying at risk patients among those exposed becomes essential. A prospective study could better determine the mechanisms of LR-MDRSE acquisition in patients managed in our orthopedic centers.

## MATERIALS AND METHODS

### Study design and setting.

The study was designed as a multicenter, retrospective, observational study of adult patients with at least one per-operative specimen positive with LR-MDRSE between 2015 and 2020. The study protocol was approved by the French Data Protection Agency (CNIL, number F20210702142117). The protocol was approved by the Clinical Ethic Group of Tours University Hospital (n° 2021 049). Written information about the study was posted in each center and the nonopposition of each patient was sought before inclusion.

### Collection of clinical data and isolates.

Recruitment was carried out in the six CRIOGO centers among patients who had orthopedic surgery between January 2015 and December 2020. Patients were identified in each center by a specific search in the database of the respective bacteriology laboratory. Microbiological and clinical characteristics of interest were collected from the patients' paper and/or computerized records. Exposure to linezolid prior to the isolation of a LR-MDRSE strain was searched for, with no limit of anteriority; thus, any linezolid therapy (600 mg ×2/d) mentioned in the medical record was considered.

### Definition of BJI.

The distinction between infection and colonization was made according to the decision of the weekly multidisciplinary meeting and the number of LR-MDRSE positive samples. In particular, all episodes treated with prolonged antibiotic therapy targeting the identified LR-MDRSE were considered infection; in case of amputation, the patient was considered infected if at least two bacteriological samples identified the same LR-MDRSE in culture. Medical records were reviewed and discussed by the authors in order to reach a retrospective consensus on infection/colonization categorization. BJI occurring on device was considered acute when symptoms had started less than 4 weeks at the time of management, and as chronic otherwise. When the date of onset of symptoms could not be retrospectively determined despite all available information in the medical record, it was by default set on the day of the surgery preceding the identification of LR-MDRSE. Patients were considered to be in failure of management in case of revision surgery for clinical failure with a new microbiological identification of the same LR-MDRSE, or unscheduled amputation or arthrodesis due to uncontrolled sepsis during follow-up, or death from any cause within 12 months after isolation of the pathogen. They were considered to be successfully managed otherwise.

### Microbiological methods.

All specimens were cultured according to the method previously described (36), and antimicrobial susceptibility testing was performed according to CASFM/EUCAST recommendations, edition April 2021 (23). Resistance to linezolid identified by the routine method was confirmed by measuring linezolid MIC using the Etest (bioMérieux) method on Mueller-Hinton agar plates (23). MICs for ceftaroline, ceftobiprole, daptomycin, dalbavancin, tigecycline, eravacycline, and delafloxacin were determined by the Etest method (23). MICs were interpreted using CASFM/EUCAST criteria, and when no breakpoint existed for S. epidermidis, S. aureus criteria were used (ceftaroline ≤1/>2mg/L; ceftobiprole ≤2/>2mg/L, delafloxacin ≤0.25/>0.25 mg/L, eravacycline ≤0.25/>0.25 mg/L) (23).

### Genetic analysis.

A total of 44/46 isolates were sent to the French National Center for *Staphylococci* (FNRCS) for expertise (for more details see reference 22).

**(i) Genetic determinants of linezolid resistance**. Genetic determinants of linezolid resistance were assessed by whole-genome sequencing (WGS) (22).

**(ii) WGS.** Sequencing libraries were generated with the Nextera DNA Prep kit (Illumina, San Diego, CA, US). WGS was performed with an Illumina instrument to generate 300 bp paired-end reads. Raw reads were trimmed using Trimmomatic v0.32 (37) then used for *de novo* assemblies with SPAdes v3.14. WGS data from all samples sequenced in this study are available (project accession PRJEB54676 [ENA]).

**(iii) *In silico* analysis**. The MLST of S. epidermidis isolates was performed from assembled genomes using *mlst* (https://github.com/tseemann/mlst). The search for acquired *cfr* linezolid resistance gene, as well as point mutations in 23S rRNA, ribosomal proteins, *rpoB,* and genes coding for the targets of fluoroquinolones was performed (22).

**(iv) Single nucleotide polymorphism and phylogenetic analysis.** The reads data of 328 S. epidermidis ST2 isolates (strains from the present study), strains described by Lee et al. (20), and other SE ST2 strains from the FNRCS collection were used to construct a phylogeny of the ST2 genetic background. Variant calling and core-genome SNP were produced using snippy v 4.6.0 (https://github.com/tseemann/snippy) and the genome of strain BHP0662 as reference. Potential recombination events were removed using gubbins (38). The phylogenetic tree based on core-genome SNPs was constructed using FastTree 2,31 (22). Tree visualization was performed using iTOL (39).
