# Supplementary material for: Clinical, Bacteriological, and Genetic Characterization of Bone and Joint Infections Involving Linezolid-Resistant Staphylococcus epidermidis: a Retrospective Multicenter Study in French Reference Centers
Source: Microbiol Spectr. 2023 May 3;11(3):e04190-22. doi: 10.1128/spectrum.04190-22 (PMC10269892; doi:10.1128/spectrum.04190-22)
Supplement: Supplemental file 1 — Table S1; Table S2. Download spectrum.04190-22-s0001.pdf, PDF file, 0.5 MB [file spectrum.04190-22-s0001.pdf]

Supplementary Table:

Table S1: Surgical management of the 46 LR-MDRSE infected or colonized patients.

Surgical management *:*	Total<br>N (%)	Infection<br>N (%)	Colonization<br>N (%)
- Complete implant exchange or ablation	21 (45.7)	18 (50.0)	3 (30.0)
◦ One-stage prosthesis exchange	8 (17.4)	8 (22.2)	0 (0)
◦ Two-stage prosthesis exchange	2 (4.3)	2 (5.6)	0 (0)
◦ Second stage of prosthesis exchange	3 (6.5)	2 (5.6)	1 (10.0)
◦ Material removal, no implantation	3 (6.5)	2 (5.6)	1 (10.0)
◦ Material removal, arthrodesis	3 (6.5)	2 (5.6)	1 (10.0)
◦ Second stage of Masquelet procedure	1 (2.2)	1 (2.8)	0 (0)
◦ Spacer exchange	1 (2.2)	1 (2.8)	0 (0)
- Debridement Antibiotics, and Implant Retention (DAIR)	10 (21.7)	9 (25.0)	1 (10.0)
- Debridement without device	8 (17.4)	5 (13.9)	3 (30.0)
◦ No per-procedure implantation	6 (13.0)	4 (11.1)	2 (20.0)
◦ Per-procedure arthrodesis	2 (4.3)	1 (2.8)	1 (10.0)
- Surgically maintained fistula	1 (2.2)	1 (2.8)	0 (0)
- Amputation	3 (6.5)	2 (5.6)	1 (10.0)
- Medical management only	3 (6.5)	1 (2.8)	2 (20.0)
Success	-	26 (72.2)	-
Total	46 (100.0)	36 (100.0)	10 (100.0)

Supplementary Table:

<u>Table S2</u>: Pathogens associated with LR-MDRSE bone and joint infections.

Characteristics	Total<br>N (%)	Infection<br>N (%)	Colonization<br>N (%)
Other associated pathogen(s)	**27 (58,7)**	21 (58,3)	6 (60,0)
- *S. aureus*	2 (4,3)	1 (2,8)	1 (10,0)
- Coagulase negative staphylococcus (other)	4 (8,7)	3 (8,3)	1 (10,0)
- *E. faecalis*	3 (6,5)	1 (2,8)	2 (20,0)
- *C. tuberculostearicum*	3 (6,5)	3 (8,3)	0 (0)
- *E. cloacae/aerogenes*	4 (8,7)	3 (8,3)	1 (10,0)
- Other enterobacteriaceae	5 (10,9)	4 (11,1)	1 (10,0)
- *P. aeruginosa*	4 (8,7)	2 (5,6)	2 (20,0)
- *C. acnes*	3 (6,5)	3 (8,3)	0 (0)
- Other bacterial species	4 (8,7)	2 (5,6)	2 (20,0)
- *C. albicans*	2 (4,3)	2 (5,6)	0 (0)
Total	46 (100)	36 (100)	10 (100)

A total of 5 patients (3 infected and 2 colonized) had 3 or more different specimens on culture-positive samples.
